# Detection of Anti-Erythrocyte Antibodies in Dogs with Inflammatory Bowel Disease (IBD)

**DOI:** 10.3390/ani11092580

**Published:** 2021-09-02

**Authors:** Eleonora Gori, Alessio Pierini, Martina Nesci, Elena Benvenuti, Silvia Tasca, George Lubas, Veronica Marchetti

**Affiliations:** 1 Veterinary Teaching Hospital “Mario Modenato”, Department of Veterinary Sciences, University of Pisa, 56124 Pisa, Italy; eleonora.gori@vet.unipi.it (E.G.); martinanesci11@gmail.com (M.N.); elebenve81@gmail.com (E.B.); george.lubas@unipi.it (G.L.); veronica.marchetti@unipi.it (V.M.); 2 Clinical Pathology Division, San Marco Veterinary Clinic and Laboratory, 35030 Padova, Italy; silvia.tasca@sanmarcovet.it

**Keywords:** enteropathy, canine, flow cytometry, hematology

## Abstract

**Simple Summary:**

In human patients with intestinal inflammation, many other diseases may occur due to it, but their presence in dogs is rarely described. The aim of the study was to prove if antibodies against red blood cells (RBC) were present in dogs with chronic intestinal inflammation (IBD), as it is demonstrated for humans, and to described other RBC abnormalities. IBD was diagnosed using the current consensus and a disease severity score was assessed for each dog, as well as score from endoscopy and intestinal histology. Our results showed that most of dogs had antibodies against RBC and 50% of dogs showed RBC regeneration in addition to chronic inflammation hematologic findings. In IBD dogs, the presence of anti-RBC antibodies and the other RBC alterations may suggest a subclinical RBC damaged (hemolysis).

**Abstract:**

Several extra-intestinal manifestations, including immune-mediated cytopenias, are reported in human inflammatory bowel disease (IBD), whereas they are poorly documented in dogs. Hypothesizing that immune-mediated subclinical anemia can occur in canine IBD, the study aim was to evaluate the erythrogram and the presence of anti-RBC antibodies in dogs with IBD. IBD was diagnosed according to the following criteria: chronic gastrointestinal signs, ruling out of extra-intestinal diseases, no improvement with diet trial, histological evidence of inflammatory infiltration, and improvement after immunosuppressant therapy. Canine Chronic Enteropathy Clinical Activity Index (CCECAI) endoscopic and histopathological scores were assessed for each dog. Twenty-five dogs were enrolled, and each dog had a CBC evaluation prior to endoscopy. The CBC was performed using laser hematology analyzer and blood smears were carefully reviewed for the presence of nucleated RBC, anisocytosis, polychromasia, and Howell–Jolly bodies. IgG and IgM anti-RBC antibodies were evaluated with flow cytometry. A high frequency of positive cases for anti-RBC antibodies in dogs with IBD (17/25 dogs) was ascertained. Approximatively 50% of dogs showed some hematologic features of RBC regeneration in addition to hematologic findings consistent with chronic inflammation. Anti-RBC antibodies and signs of erythroid regeneration may suggest possible subclinical chronic immune-mediated hemolysis that can cause anemia in dogs with IBD, together with the chronic inflammation.

## 1. Introduction

Extraintestinal manifestations (EIMs) are defined as “symptoms that occur in the human patient with inflammatory bowel disease (IBD) and that arise from the involvement of other organs or systems by extension/translocation of the inflammatory process or by an independent but perpetuated event from IBD or that shares environmental or genetic predisposition with it” [1]. The pathogenesis of EIMs in IBD is believed to be linked to immune response itself caused by the intestinal disease. Otherwise, extraintestinal complications are mainly caused by the disease itself (e.g., malabsorption with consequent micronutrient deficiencies, osteoporosis, peripheral neuropathies, kidney stones, gallstones, and IBD drug-related side effects) [2]. In veterinary medicine, this distinction is not as clear as it is in human medicine.

In human IBD, EIMs are well documented, with a prevalence ranging from 31% in patients with Crohn’s disease and 43% in patients with ulcerative colitis [2]. Musculoskeletal and dermatologic manifestations are the most common, followed by hepato-pancreatic, ocular, renal, pulmonary [3], and hematopoietic manifestations [1]. IBD and EIMs may share a common pathogenic pathway, since one EIM increases the probability of another EIM and because they tend to appear simultaneously [4]. EIMs can arise from intestinal inflammation through various mechanisms, such as aberrant lymphocyte activity. EIMs may also develop from a proinflammatory state with the increase of specific cyto- and chemokines [4]. Most probably, different specific mechanisms are involved, which are not mutually exclusive [4]. In recent years, accumulating evidence especially on oxidative stress in the pathogenesis and progression of IBD is increasing [5,6]. The presence of reactive oxygen species and antioxidant defense in the gastrointestinal tract, oxidative stress involvement in the start and progression of IBD and its relationships with genetic susceptibility and mucosal immune response has been demonstrated [5,6]. However, even if substantial progress in understanding the role of oxidative stress in IBD in humans and experimental animals has been made, the underlying mechanisms are still not well defined [5,6].

Autoimmune cytopenias have been reported in human patients with IBD with a low prevalence (0.2–1.7%), which rises to 28% in ulcerative colitis [7]. In both human and veterinary literature, there is no mention of anti-erythrocyte antibodies outside the studies on immune-mediated hemolytic anemia (IMHA), especially associated with ulcerative colitis [7,8,9,10,11,12]. Although the overall prevalence of IMHA is low, it appears higher in patients with IBD than in the general population [7]. In veterinary medicine, there are few studies on hematological EIMs in gastroenteric diseases. IMHA has been described in both dogs and cats with pancreatitis, in which an association has been established [13,14,15,16]. In dogs, IBD is defined as an idiopathic, multifactorial intestinal inflammation, in which diet and antibiotic trials have failed [17]. In addition, in IRE, intestinal inflammation has to be demonstrated by histopathology and a response to an immunosuppressant/steroid therapy [17,18]. We hypothesized that dogs with IBD may have a subclinical form of immune-mediated anemia or at least the presence of anti-RBC antibodies secondary to the gastrointestinal inflammatory process, as it is for human patients. In addition, we hypothesized that the more severe IBD indices, the more frequent the presence of anti-RBC antibodies. The objective of the study was to describe the frequency and levels of anti-RBC antibodies in a population of IBD dogs and to evaluate the association between both clinical and hematological parameters and the presence/absence of anti-RBC antibodies.

## 2. Materials and Methods

This prospective research was conducted on canine patients diagnosed with IBD presented at the Veterinary Teaching Hospital “Mario Modenato” of the University of Pisa. Blood samples required for hematological tests were taken after obtaining the owners’ informed consent, and the study received the official approval of the animal welfare committee of the University of Pisa (OPBA number 31834/2017).

Dogs included in the study had chronic gastrointestinal disease (including weight loss, vomiting, diarrhea, decreased appetite) for at least three weeks. Each dog had full hematological examination and a biochemical panel including serum trypsin-like immunoreactivity, canine pancreatic lipase, serum cobalamin, serum basal cortisol, and urinalysis including urinary protein-to-creatinine ratio and abdominal ultrasound performed at the time of the inclusion. Extra-intestinal disease or non-primary inflammatory intestinal disease (e.g., intussusception, foreign bodies, or intestinal tumors) were excluded. Food and antibiotic-responsive enteropathies were excluded before endoscopy if responding either to hydrolyzed or monoprotein diet trial with an added multistrain probiotic for at least of 2 weeks, or to an antibiotic trial with tylosin at 15 mg/kg every 12 h for 3 weeks, respectively [17,18,19]. Dogs treated with corticosteroids or other immunomodulatory drugs within two months prior admission were also excluded. All dogs underwent abdominal ultrasound and thoracic radiography to rule-out clinical conditions i.e., neoplasia that could rise possible positivity do anti-RBC antibodies.

In all dogs, at the time of the upper and lower gastrointestinal endoscopy, the clinical severity was evaluated using the previously published and validated Chronic Canine Enteropathy Clinical Activity Index (CCECAI) score [20]. All dogs underwent gastrointestinal endoscopy and biopsy samples were collected to perform a histopathological examination according to World Small Animal Veterinary Association (WSAVA) Gastrointestinal Standardization Group [20,21]. Intestinal biopsies were performed by endoscopy using 2.4 mm biopsy forceps, depending on the patients’ size. At least eight samples were collected from each of the following sites: stomach, duodenum, colon, and ileum (when available) (Fujinon EG-200FP, Fujinon Corporation, Saitama, Japan). Endoscopic and histopathological grading of the duodenum were performed in each dog according to WSAVA guidelines [21,22]. Only duodenal endoscopic and histological scores were recorded because severe mucosal lesions in the duodenum, besides other intestinal sites alterations, were significantly associated with negative outcome in dogs with CE [20]. In addition, all dogs had duodenum evaluation, contrarily to the ileum. The endoscopic score of the duodenum was recorded ranging from normal (0), mild (1), moderate (2), to severe (3), whereas the histopathological score for duodenum ranged from normal (0), mild (1), moderate (2), to severe (3).

Just before the endoscopic procedure, a blood sample (2 mL) was collected in test tubes containing K3-EDTA from each dog to perform the complete blood count and the flow cytometry for the detection of anti-RBC antibodies. All blood samples were immediately processed and double-blinded for evaluation at the Clinical Pathology Laboratory of the University of Pisa for the blood count within 4 h from the collection (IDEXX Procyte Dx^®^, IDEXX Laboratories, Milan, Italy). Blood smears were stained with an automatic slide stainer (Aerospray Wescor, Delcon, Milan, Italy) using a May–Grünwald–Giemsa staining and evaluated by an experienced clinical pathologist (G.L.). Presence or absence of erythrocyte cytomorphological alterations were recorded and poikilocytosis were examined using a 4-grade scale [23]. The number of nucleated RBCs (nRBCs), if observed, were counted every 100 WBC. Dogs with a reduction in RBC count, hemoglobin (HGB), and/or hematocrit (HCT) below reference values were considered anemic. Based on the HCT, anemia was graded as mild (30–37%), moderate (29–20%), or severe (<20%) [24]. Anemia was then characterized using mean corpuscular volume (MCV, range 61–73 fL), as normo-micro or macrocytic, and mean corpuscular hemoglobin concentration (MCHC, range 32–38 g/dL), as normo- or hypochromic. The erythrocyte regeneration was assessed using the reticulocyte count and the occurrence of nRBCs, polychromasia, anisocytosis, and/or Howell–Jolly bodies.

All K3-EDTA blood samples were refrigerated after CBC analysis and transferred to an external laboratory for the flow cytometry (San Marco Veterinary Clinic, Padova, Italy) and analyzed as previously reported [25]. The anti-RBC antibodies by flow cytometry with the relative graphs and percentages of IgG values were evaluated and recorded (Beckman Coulter^®^ Cytomics FC 500). Dogs were divided into two categories based on anti-RBC antibodies positivity (positive if IgG > 0.5% or negative) [25,26].

CBC parameters (RBC, HCT, HGB, MCV, MCHC, RBC distribution width (RDW), absolute reticulocyte count) and presence of RBC abnormalities were recorded for each dog. Dogs were divided in protein-losing enteropathy (PLE)/non-PLE based on their serum albumin level (<2.7 mg/dL = PLE) [27].

The statistical analysis was performed using a commercial software (SPSS v. 23, IBM Corp., Armonk, NY, USA) and a *p*-value of <0.05 was considered statistically significant. Based on the Kolmogorov–Smirnov test for normality all continuous parameters (age, CCECAI, RBC, HCT, HGB, MCV, MCHC, RDW, absolute reticulocyte count) were non-normally distributed. Data were presented as median and range. Sex, endoscopic, and histological scores were analyzed as categorical data.

The CCECAI, RBC, HCT, HGB, MCV, MCHC, RDW, reticulocyte count, the presence of anemia, and signs of regeneration were compared between anti-RBC positive or negative groups using the Mann–Whitney U-test. A Pearson’s chi-square with z-test for column proportion comparisons and Bonferroni adjustment for multiple comparisons were calculated to test the association between anti-RBC antibodies positivity and endoscopic and histological scores, the presence of anemia, and signs of erythrocyte regeneration.

## 3. Results

A total of 25 dogs with IBD were prospectively included. Most dogs were mixed breed (6/25; 24%), followed by German Shepherds, English Setters, Springer Spaniels, and Shih Tzus (2 dogs each breed). The remaining 12 dogs belonged to the following breeds: American Staffordshire, Standard Poodle, Poodle, Bolognese, Bernese Mountain dog, Kurzhaar, Épagneul Breton, Cavalier King Charles Spaniel, Cocker Spaniel, Corso dog, and Labrador Retriever. There were 8 spayed females and 17 males (16 intact). The median age was 8 years (range 1–12 years).

The median CCECAI was 7 (range 3–13). The endoscopic score was 1 in 5 dogs (20%), 2 in 14 dogs (56%), and 3 in 6 dogs (24%). The histological score was 2 in 13 dogs (52%) and 3 in 12 dogs (48%). Based on serum albumin levels, twelve dogs (48%) were classified as PLE, whereas the other 13 as non-PLE.

**Figure 1:**
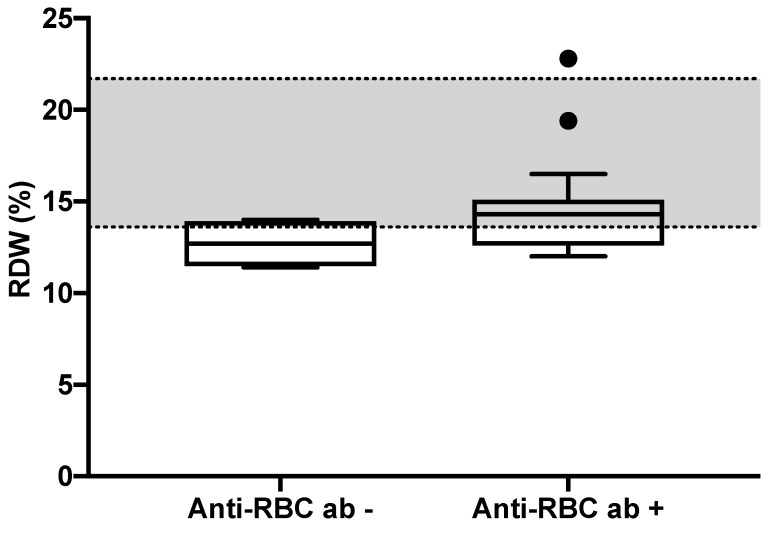
Box and whiskers of RDW (%) representation in IBD dogs with anti-RBC antibodies negative or positive (median 12.7 vs. 14.3%, *p* = 0.03). The boxes represent the 25th and 75th percentile, the line in the boxes is the median RDW value and the whiskers are minimum and maximum values. The two points in the group of anti-RBC antibodies positive are outliers. The gray band represents the RDW reference interval.

Nineteen of 25 dogs (76%) were classified as positive for the presence of anti-RBC antibodies. No association between endoscopic and histologic scores (*p* = 0.22 and *p* = 0.64, respectively), and the presence of PLE and anti-RBC antibodies positive dogs was found (*p* = 0.16). The descriptive statistics of the study population in the erythrocyte parameters is reported in Table 1. There were no differences in CCECAI, RBC, HCT, HGB, MCV, MCHC, absolute reticulocyte count between positive and negative anti-RBC antibodies dogs (*p* = 0.9, *p* = 0.12, *p* = 0.09, *p* = 0.16, *p* = 0.36, *p* = 0.33 and *p* = 0.2, respectively). Anti-RBC antibodies positive dogs had a significantly higher median RDW compared to negative dogs (14.3 vs. 12.7%, *p* = 0.03; Figure 1).

Anemia was present in 9 of 25 dogs (36%) and was classified as mild in 7 dogs and moderate in 2 cases. Anemia was microcytic in 1 case and normocytic in the remaining 8 cases and all these dogs had normochromic anemia. At the blood smears evaluation, 11 (44%) cases did not show any RBC morphology evidences. Three dogs showed RBC morphology abnormalities: One dog had only schistocytes (score +), one dog had rare keratocytes, schistocytes, and acanthocytes (all alteration score +), whereas the other dog had only rare echinocytes (+). Four dogs had Howell–Jolly bodies (two dogs +/− and the other two +) and seven dogs showed polychromasia (from +/− to ++). Finally, six dogs had nRBCs (from 1 to 4/100 WBC). Lastly, based on the presence of criteria reported above, 13 dogs had signs of RBC regeneration. No association with either the presence of anemia or erythrocyte regeneration and anti-RBC antibodies positivity were found (*p* = 0.06 and *p* = 0.07, respectively).

## 4. Discussion

This novel study investigated the presence of anti-RBC antibodies in dogs with IBD. The choice of performing anti-RBC antibodies at the same time of the gastrointestinal endoscopy was linked to the purpose of establish if there was any relation with the clinical, endoscopic, or histological scores. In addition, to avoid the biases related to immunomodulatory therapies, all the blood samples and analysis were made prior the start of IBD treatment, also excluding dogs that had immunomodulatory drugs prior inclusion. The administration of glucocorticoid drugs such as prednisone, prednisolone, methylprednisolone, or cyclosporine could hypothetically create biases in the interpretation of flow cytometry, although there are no reliable data on this aspect reported in the literature.

In human medicine, hematological changes in IBD are extensively studied because they represent frequent complications and this EIM of the disease, is contributing to the morbidity and mortality and having a significant impact on the patients’ quality of life [1,2,4,28]. Among the EIMs in human IBD both red blood cell and platelets cytopenias are described, with the development of IMHA and immune-mediated thrombocytopenia, respectively [7]. The presence of autoimmune cytopenias had higher incidence and prevalence in IBD patients than in the general population [7]. In particular, the incidence of an immune-mediated component against RBC was present in 4.1 patients out 100,000/per year with a higher prevalence in the ulcerative colitis form of IBD [7].

In veterinary medicine, extraintestinal, especially hematological, manifestations of gastrointestinal diseases are poorly studied. In acute pancreatitis, 4/12 dogs had signs of immune-mediated anemia [13] and a case-report of IMHA in a dog with pancreatitis has been reported [14]. More recently, Zoia and Drigo [16] reported a prevalence of immune-mediated anemia of 27% of cats with pancreatitis. Lastly, in 2019 a study demonstrated that dogs with immune-mediated anemia had an increased risk of developing pancreatitis, due to the free hemoglobin in the blood following the hemolytic process [15]. There are no studies that have addressed the presence for anti-RBC antibodies during chronic enteropathy.

In our study, the presence of anti-RBC antibodies was highlighted in 76% of the study population. The positivity to anti-RBC antibodies was identified by flow cytometry, and this methodology was preferred to the Coombs’ test for its greater sensitivity [29]. In fact, according to the study, the sensitivity of flow cytometry is higher (100%) than the Coombs’ test (58%) to detect anti-RBC antibodies. In contrast, specificity appears to be lower in flow cytometry than in the Coombs’ test (87.5% vs. 100%) [29,30,31]. The only comparison of this data may be found in human medicine, although the pathogenesis of an immuno-mediated anemia as EIM in humans is not entirely clear. This finding seems to be linked to cross-reactivity with microbiota and red blood cell antigens. It usually has a parallel course with IBD and is more likely to be associated with the active forms of IBD [7].

The chronic inflammation present in our population due to intestinal disease may have induced changes in the erythrocyte membrane due to oxidative stress, inducing the production of anti-erythrocyte antibodies [32]. The recent ACVIM consensus [33] suggested immune-mediated anemia may occur with other comorbidities considered to be triggers, and inflammation due to non-infectious processes was included. A generalized inflammatory state may induce the development of IMHA in dogs and cats, but there are not yet enough studies that can confirm this [33,34].

The association between the severity of the IBD (CCECAI) and the presence of PLE, as well as the endoscopic and histological scores were not associated with the presence of anti-RBC antibodies. This data may be difficult to discuss due to the novelty of the present investigation in dogs. However, in human medicine it is reported that the presence of antibodies may be concomitant, precede, or follow intestinal disease, and this is particularly true in patients with ulcerative colitis [7].

In our study, anemia was found in 36% of IBD dogs. These data are only partially in agreement with with Craven and colleagues, who reported mild anemia, affecting 12% of canine patients with chronic intestinal inflammatory disease [34,35].

Anemia in human IBD patients, reported in approximately 24% of cases, is more commonly due to an iron deficiency anemia or chronic inflammatory anemia (anemia of chronic disease). Other less common causes of anemia during IBD are cobalamin and folate deficiency, drug-induced anemia, and hemolytic non-immunomediated anemia [1,28].

Anemia was then characterized, based on HCT and RBC indices, to evaluate its possible pathogenetic mechanism. We found that normocytic normochromic anemia was the most frequent type (8/9), followed by microcytic normochromic anemia (1/9). It may be hypothesized that that the main mechanism may be linked to an anemia of chronic disease. This hypothesis is supported by the study of Marchetti et al. [36] as the main pattern of anemia of chronic disease was normocytic normochromic anemia. This type of anemia was mainly caused by reduced absorption of iron in the intestine and decreased release from the reticuloendothelial system due to hepcidin overexpression. Secondly, reduced release of renal erythropoietin resulting in decreased erythropoiesis or an “erythropoietin resistance”, due to the downregulation of erythropoietin receptors. Finally, a decreased proliferation and defective maturation of erythrocyte precursors or reduced life span of erythrocytes due to intense splenic erythrophagocytosis [28]. However, the possibility of an iron deficiency anemia was not investigated in our study as the possible presence of fecal occult blood, as well as the iron profile (serum iron, ferritin, and transferrin), were not included. It is interesting in our opinion to note that of these nine anemic subjects, all of them tested positive for the presence of anti-RBC antibodies, and that one in two cases that presented microcytic anemia also displayed some signs of erythrocyte regeneration. In fact, these two dogs had both increased reticulocyte count (123 and 199 K/μL), increased RDW and polychromasia, anisocytosis, presence of nRBCs, and Howell–Jolly bodies at the blood smear evaluation. Among anti-RBC antibodies positive dogs, some signs of erythrocyte regeneration were present in 35% of dogs. Due to these findings, it is not possible to exclude an increased destruction (hemolysis), even if subclinical, due to activation of the humoral immune system could have occur. Indeed, in a veterinary review on the diagnostic approach to anemia reported that hemolytic anemia may have a pre-regenerative phase (normochromic normocytic) and later become hypochromic macrocytic [37]. Inflammatory cytokines and oxidative damage, released during the inflammatory process, can induce changes on the erythrocyte membrane by damaging red blood cells and inducing the production of anti-erythrocyte antibodies [38]. Nevertheless, it is important to consider the hypothesis that the pathogenesis of anemia during IBD may be multifactorial, not excluding the possibility that there may be an overlap and diversity of anemia patterns.

The examination of the blood smear can help us to characterize anemia with morphological abnormalities. In our study, changes in normal erythrocyte morphology on the smear were seen in 56% of the total population examined. The alterations found most frequently were polychromasia (28%), nRBCs (24%), and anisocytosis (20%). The frequency of signs of erythrocyte regeneration increases, if we consider anti-RBC antibodies positive patients (12/25, 63.2%). In fact, also in the study by Marchetti and colleagues [36], anisocytosis (59.0%), polychromasia (40.9%), and Howell–Jolly bodies (13.6%) were reported in the blood smear. These features usually do not belong to chronic disease anemia but are more frequently associated with regenerative anemias [22].

Another interesting result is the presence of nRBCs, which are not normally present in chronic patients, especially if not anemic [22]. In our dogs, the finding of few nRBCs in the peripheral blood may be caused by a blood–marrow barrier damage, possibly caused by hypoxia or, alternatively, due to hematopoiesis in response to anemia [39]. The possible slow or intermittent blood loss in the gastrointestinal tract may also be the cause of this overlap of chronic and regenerative signs of the anemia. The presence of fecal occult blood has not been investigated, therefore, although none of the cases clinically had clinical melena or hematochezia, it cannot be ruled out. Another hypothesis that could justify these regenerative signs may be a subclinical hemolytic process with a possible immune-mediated basis, which may be indicated by the high percentage of dogs with anti-RBC antibodies.

We also highlighted that dogs with anti-RBC antibodies had a significantly higher RDW than dogs that did not show antibodies. These data are, hypothetically, consistent with a possible subclinical hemolytic process, which may be responsible of erythrocyte signs of regeneration such as anisocytosis, and thus a higher RDW. This finding may be compared with human medicine, in which RDW has be thoroughly studied and recently reassumed in a review as a hematological marker in human Crohn’s disease and ulcerative colitis [40]. A higher RDW has been related to disease activity in both Crohn’s disease and ulcerative colitis patients [40]. Various pathogenic mechanisms were proposed: The most probable is connected to anemia, and others are inflammation and oxidative stress on the normal process of erythropoiesis [40].

This study has several limitations. Firstly, the choice to perform this study of IBD dogs limited the population size. In fact, the IBD diagnosis, being a step-by-step diagnosis, requires a longer timeframe than other intestinal conditions [17,18,19]. Another limitation that reduced the sample size was the difficulty of finding dogs without co-morbidities and the lack of a control group. Another limitation is linked to the high percentage of positive anti-RBC antibodies dogs (19/25 dogs), which may influence the absence of some statistically significant associations. Lastly, it would be useful and interesting to repeat the flow-cytometry during the follow-up of dogs to investigate how the clinical severity and response to therapy may affect the anti-RBC antibodies positivity course.

## 5. Conclusions

To our knowledge, this is the first study in veterinary medicine that evaluated the presence of anti-RBC antibodies in dogs with IBD. Our work highlighted a high percentage of anti-RBC antibodies positive cases in the study population. Furthermore, in all the anemic dogs, anti-RBC antibodies were detected.

In IBD dogs, a combination of anemia of chronic disease and immune-mediated hemolytic mechanisms may coexist. Although, the pathogenetic mechanisms underlying anemia in during IBD may be multiple and overlapping. Further studies and larger population size will allow a more in-depth evaluation of the presence of iron deficiency anemia could allow us to better understand the role of anti-RBC antibodies in anemia.

Even if we were only looking at anti-RBC antibodies and not an IMHA, which is a complex disease with a precise classification, it seems that a connection between intestinal inflammation and the presence of anti-erythrocyte antibodies may exist.

## Figures and Tables

**Table 1:** Descriptive statistics of erythrocyte parameters in the study population.

Parameter	All Dogs	Anti-RBC ab ⊖	Anti-RBC ab ⊕	Reference Range
RBC (×10^6^/μL)	6 (3.8–7.8)	6.6 (5.5–7.8)	6 (3.8–7.8)	5.65–8.8
HCT (%)	42.5 (23.6–57.5)	47.3 (37.3–54)	39.9 (23.6–57.5)	37.3–61.7
HGB (g/dL)	14.4 (8.5–20)	15.4 (13.5–19)	13.5 (8.5–20)	13.1–20.5
MCV (fL)	68.6 (57–77.1)	69 (67.9–75.2)	67.7 (57–77.1)	61–73
MCHC (g/dL)	34.6 (31.6–37.2)	33.9 (31.6–36.2)	34.8 (32–37.2)	32–38
RDW (%)	14 (11.4–22.8)	12.7 (11.4–14)	14.3 (12–22.8) ^1^	13.6–21.7
Retic (×10^3^/μL)	35 (13–361)	32 (13–61)	40 (14–361)	10–110

⊖, negative; ⊕, positive; RBC, red blood cell count; HCT, hematocrit; HGB, hemoglobin; MCV, mean corpuscular volume; MCHC, mean corpuscular hemoglobin concentration; RDW, RBC distribution width; Retic, reticulocyte count. ^1^ *p* = 0.03 (Mann–Whitney U-test).

## Data Availability

The data presented in this study are available on request from the corresponding author. The data are not publicly available due to other project involvement.
